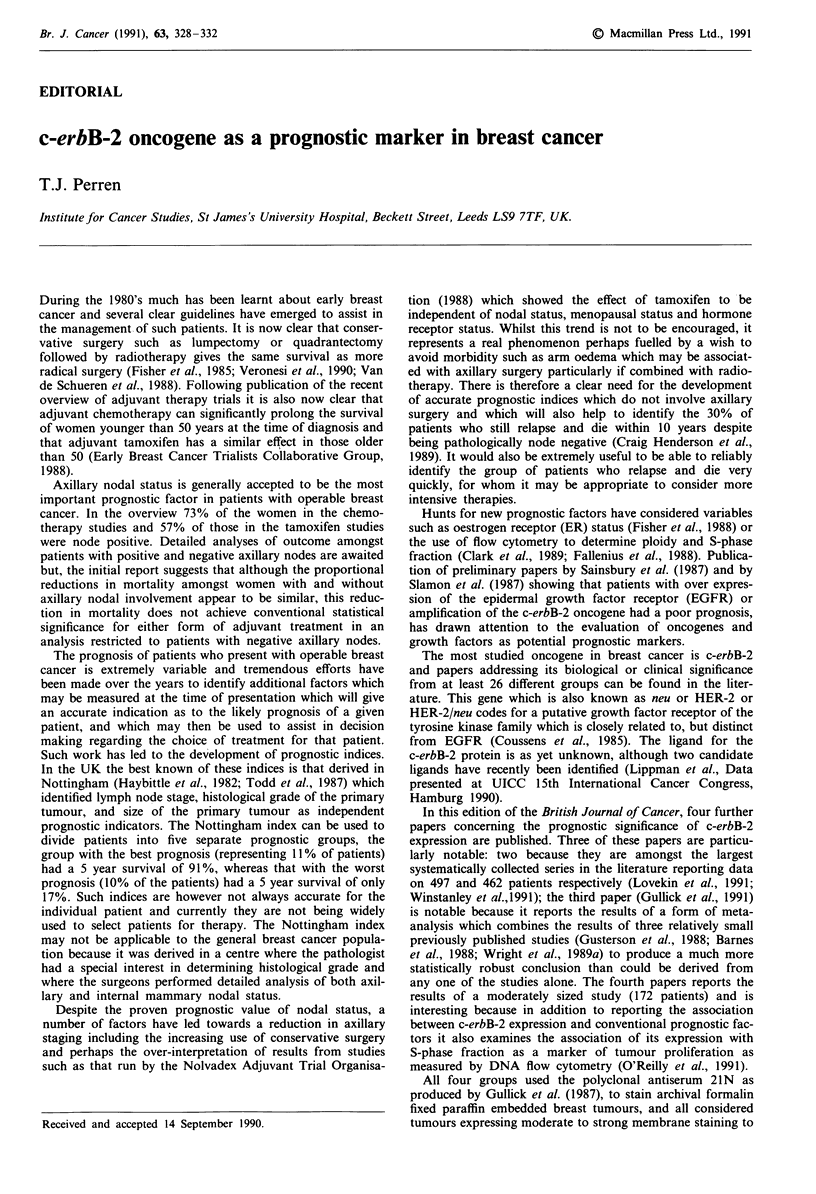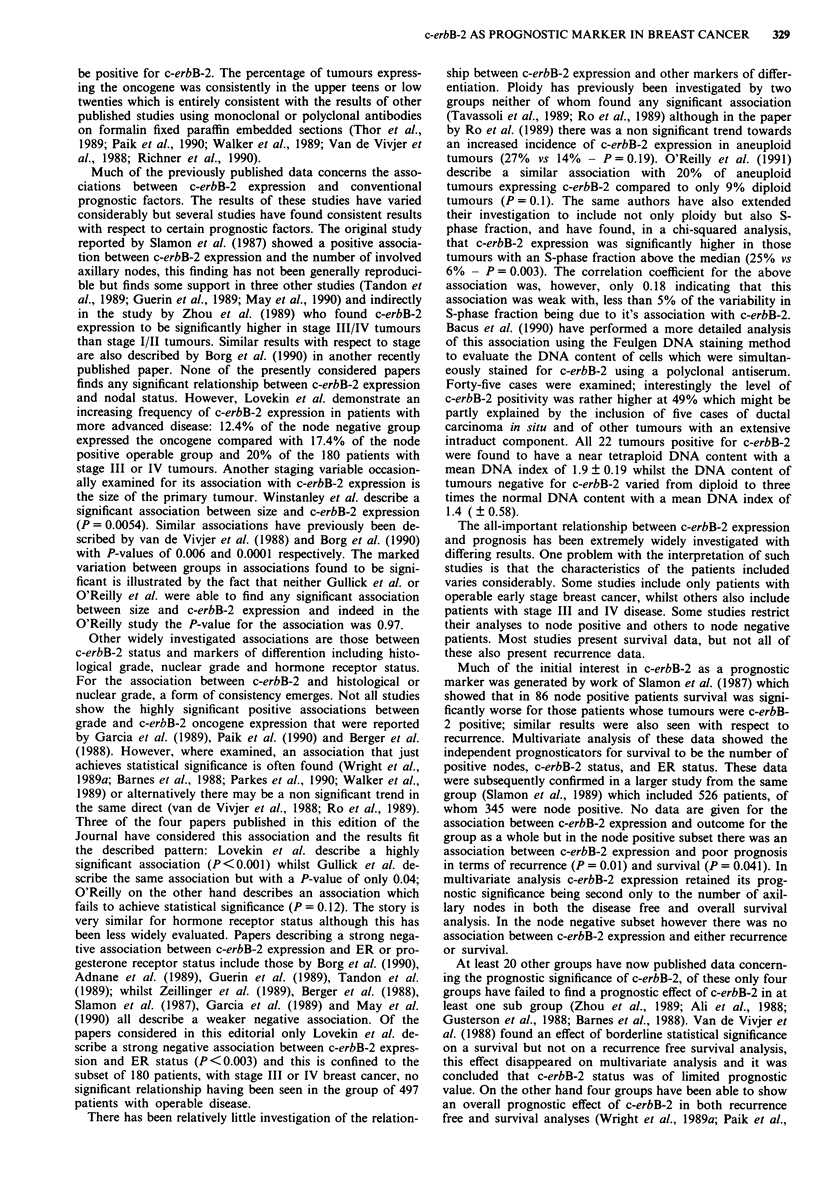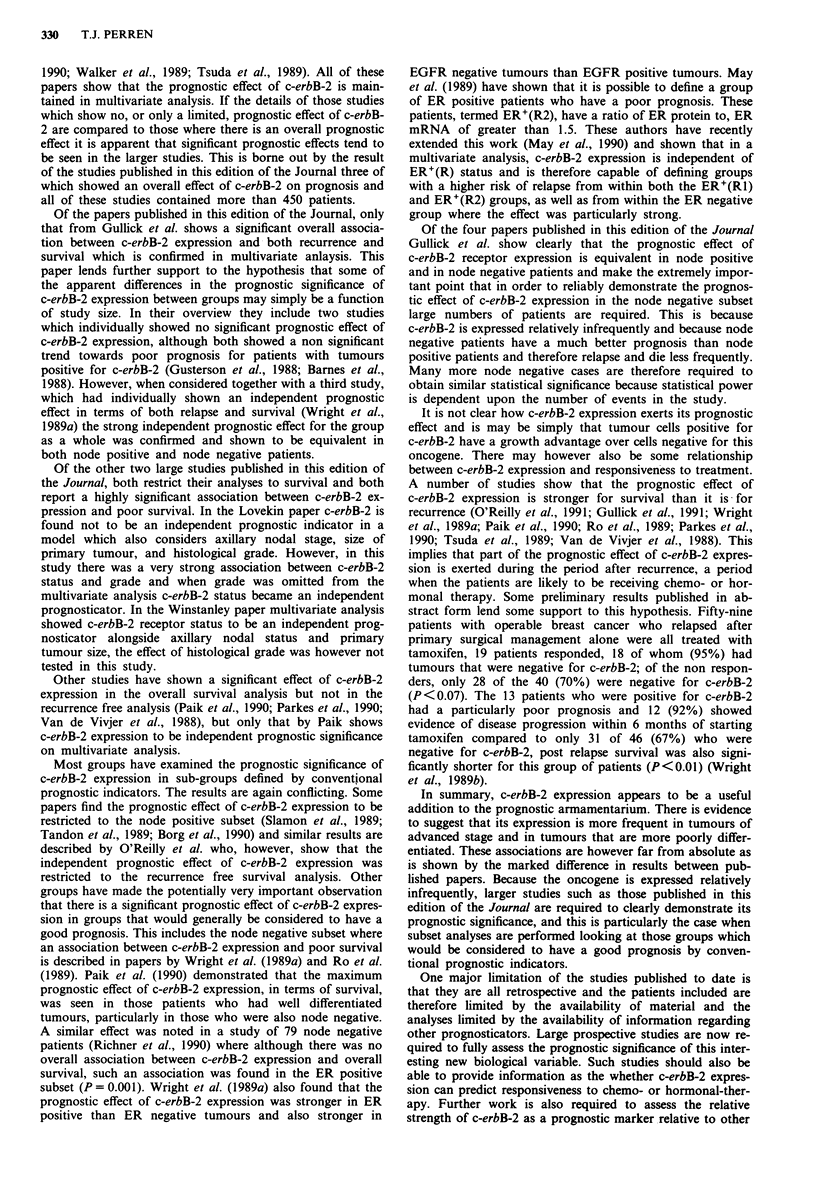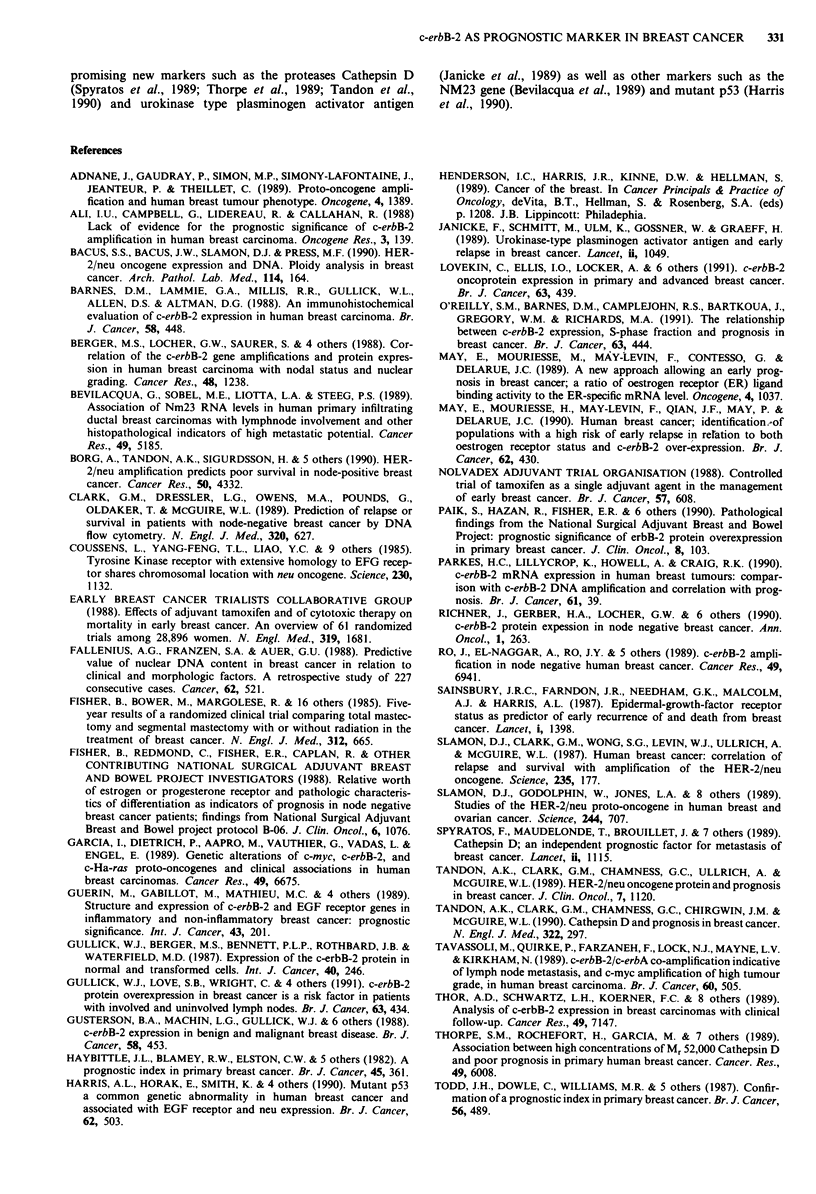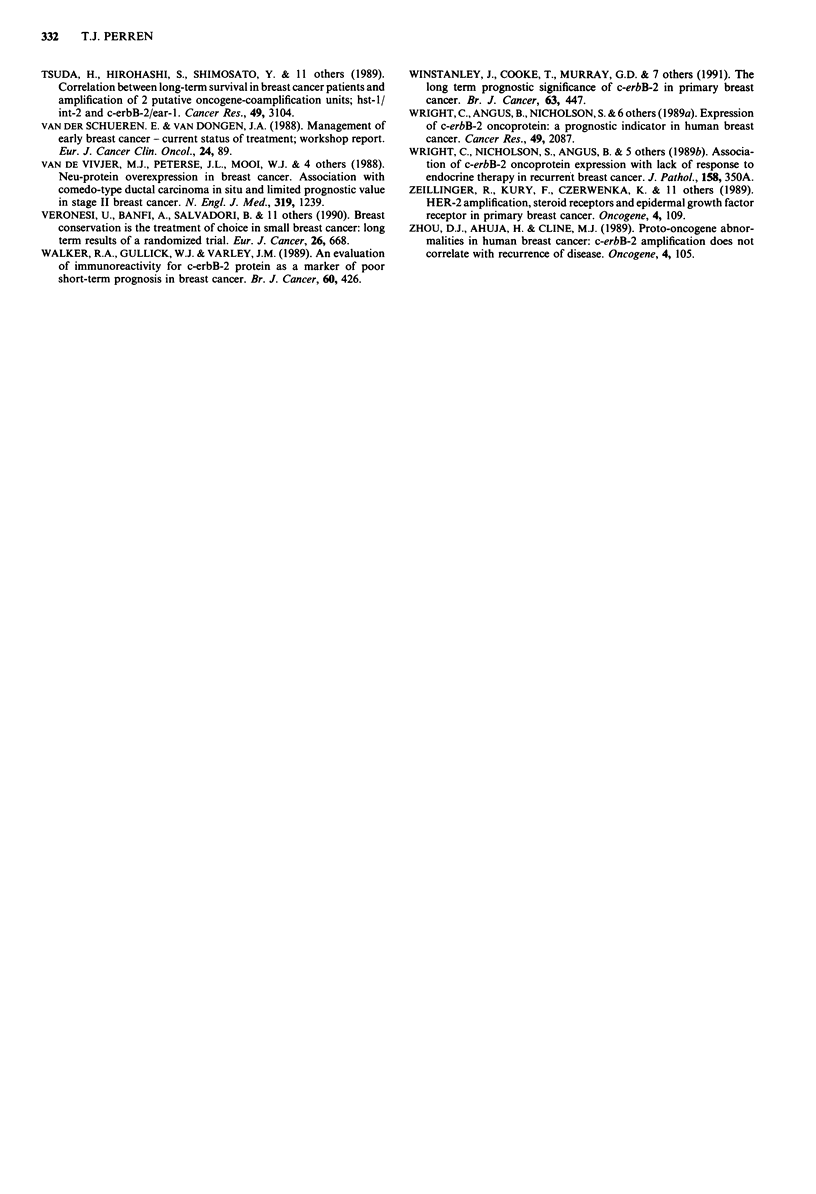# c-erbB-2 oncogene as a prognostic marker in breast cancer.

**DOI:** 10.1038/bjc.1991.78

**Published:** 1991-03

**Authors:** T. J. Perren


EDITORIAL

### c-*erb*B-2 oncogene as a prognostic marker in breast cancer

T.J. Perren

*Institute for Cancer Studies, St James's University Hospital, Beckett Street, Leeds LS9 7TF, UK.*

During the 1980's much has been learnt about early breast cancer and several clear guidelines have emerged to assist in the management of such patients. It is now clear that conservative surgery such as lumpectomy or quadrantectomy followed by radiotherapy gives the same survival as more radical surgery (Fisher *et al.*, 1985; Veronesi *et al.*, 1990; Van de Schueren *et al.*, 1988). Following publication of the recent overview of adjuvant therapy trials it is also now clear that adjuvant chemotherapy can significantly prolong the survival of women younger than 50 years at the time of diagnosis and that adjuvant tamoxifen has a similar effect in those older than 50 (Early Breast Cancer Trialists Collaborative Group, 1988).

Axillary nodal status is generally accepted to be the most important prognostic factor in patients with operable breast cancer. In the overview 73% of the women in the chemotherapy studies and 57% of those in the tamoxifen studies were node positive. Detailed analyses of outcome amongst patients with positive and negative axillary nodes are awaited but, the initial report suggests that although the proportional reductions in mortality amongst women with and without axillary nodal involvement appear to be similar, this reduction in mortality does not achieve conventional statistical significance for either form of adjuvant treatment in an analysis restricted to patients with negative axillary nodes.

The prognosis of patients who present with operable breast cancer is extremely variable and tremendous efforts have been made over the years to identify additional factors which may be measured at the time of presentation which will give an accurate indication as to the likely prognosis of a given patient, and which may then be used to assist in decision making regarding the choice of treatment for that patient. Such work has led to the development of prognostic indices. In the UK the best known of these indices is that derived in Nottingham (Haybittle *et al.*, 1982; Todd *et al.*, 1987) which identified lymph node stage, histological grade of the primary tumour, and size of the primary tumour as independent prognostic indicators. The Nottingham index can be used to divide patients into five separate prognostic groups, the group with the best prognosis (representing 11% of patients) had a 5 year survival of 91%, whereas that with the worst prognosis (10% of the patients) had a 5 year survival of only 17%. Such indices are however not always accurate for the individual patient and currently they are not being widely used to select patients for therapy. The Nottingham index may not be applicable to the general breast cancer population because it was derived in a centre where the pathologist had a special interest in determining histological grade and where the surgeons performed detailed analysis of both axillary and internal mammary nodal status.

Despite the proven prognostic value of nodal status, a number of factors have led towards a reduction in axillary staging including the increasing use of conservative surgery and perhaps the over-interpretation of results from studies such as that run by the Nolvadex Adjuvant Trial Organisation (1988) which showed the effect of tamoxifen to be independent of nodal status, menopausal status and hormone receptor status. Whilst this trend is not to be encouraged, it represents a real phenomenon perhaps fuelled by a wish to avoid morbidity such as arm oedema which may be associated with axillary surgery particularly if combined with radiotherapy. There is therefore a clear need for the development of accurate prognostic indices which do not involve axillary surgery and which will also help to identify the 30% of patients who still relapse and die within 10 years despite being pathologically node negative (Craig Henderson *et al.*, 1989). It would also be extremely useful to be able to reliably identify the group of patients who relapse and die very quickly, for whom it may be appropriate to consider more intensive therapies.

Hunts for new prognostic factors have considered variables such as oestrogen receptor (ER) status (Fisher *et al.*, 1988) or the use of flow cytometry to determine ploidy and S-phase fraction (Clark *et al.*, 1989; Fallenius *et al.*, 1988). Publication of preliminary papers by Sainsbury *et al.* (1987) and by Slamon *et al.* (1987) showing that patients with over expression of the epidermal growth factor receptor (EGFR) or amplification of the c-*erb*B-2 oncogene had a poor prognosis, has drawn attention to the evaluation of oncogenes and growth factors as potential prognostic markers.

The most studied oncogene in breast cancer is c-*erb*B-2 and papers addressing its biological or clinical significance from at least 26 different groups can be found in the literature. This gene which is also known as *neu* or HER-2 or HER-2/*neu* codes for a putative growth factor receptor of the tyrosine kinase family which is closely related to, but distinct from EGFR (Coussens *et al.*, 1985). The ligand for the c-*erb*B-2 protein is as yet unknown, although two candidate ligands have recently been identified (Lippman *et al.*, Data presented at UICC 15th International Cancer Congress, Hamburg 1990).

In this edition of the *British Journal of Cancer*, four further papers concerning the prognostic significance of c-*erb*B-2 expression are published. Three of these papers are particularly notable: two because they are amongst the largest systematically collected series in the literature reporting data on 497 and 462 patients respectively (Lovekin *et al.*, 1991; Winstanley *et al.*,1991); the third paper (Gullick *et al.*, 1991) is notable because it reports the results of a form of meta-analysis which combines the results of three relatively small previously published studies (Gusterson *et al.*, 1988; Barnes *et al.*, 1988; Wright *et al.*, 1989*a*) to produce a much more statistically robust conclusion than could be derived from any one of the studies alone. The fourth papers reports the results of a moderately sized study (172 patients) and is interesting because in addition to reporting the association between c-*erb*B-2 expression and conventional prognostic factors it also examines the association of its expression with S-phase fraction as a marker of tumour proliferation as measured by DNA flow cytometry (O'Reilly *et al.*, 1991).

All four groups used the polyclonal antiserum 21N as produced by Gullick *et al.* (1987), to stain archival formalin fixed paraffin embedded breast tumours, and all considered tumours expressing moderate to strong membrane staining to

Received and accepted 14 September 1990.

be positive for c-*erb*B-2. The percentage of tumours expressing the oncogene was consistently in the upper teens or low twenties which is entirely consistent with the results of other published studies using monoclonal or polyclonal antibodies on formalin fixed paraffin embedded sections (Thor *et al.*, 1989; Paik *et al.*, 1990; Walker *et al.*, 1989; Van de Vivjer *et al.*, 1988; Richner *et al.*, 1990).

Much of the previously published data concerns the associations between c-*erb*B-2 expression and conventional prognostic factors. The results of these studies have varied considerably but several studies have found consistent results with respect to certain prognostic factors. The original study reported by Slamon *et al.* (1987) showed a positive association between c-*erb*B-2 expression and the number of involved axillary nodes, this finding has not been generally reproducible but finds some support in three other studies (Tandon *et al.*, 1989; Guerin *et al.*, 1989; May *et al.*, 1990) and indirectly in the study by Zhou *et al.* (1989) who found c-*erb*B-2 expression to be significantly higher in stage III/IV tumours than stage I/II tumours. Similar results with respect to stage are also described by Borg *et al.* (1990) in another recently published paper. None of the presently considered papers finds any significant relationship between c-*erb*B-2 expression and nodal status. However, Lovekin *et al.* demonstrate an increasing frequency of c-*erb*B-2 expression in patients with more advanced disease: 12.4% of the node negative group expressed the oncogene compared with 17.4% of the node positive operable group and 20% of the 180 patients with stage III or IV tumours. Another staging variable occasionally examined for its association with c-*erb*B-2 expression is the size of the primary tumour. Winstanley *et al.* describe a significant association between size and c-*erb*B-2 expression ($P = 0.0054$). Similar associations have previously been described by van de Vivjer *et al.* (1988) and Borg *et al.* (1990) with *P*-values of 0.006 and 0.0001 respectively. The marked variation between groups in associations found to be significant is illustrated by the fact that neither Gullick *et al.* or O'Reilly *et al.* were able to find any significant association between size and c-*erb*B-2 expression and indeed in the O'Reilly study the *P*-value for the association was 0.97.

Other widely investigated associations are those between c-*erb*B-2 status and markers of differention including histological grade, nuclear grade and hormone receptor status. For the association between c-*erb*B-2 and histological or nuclear grade, a form of consistency emerges. Not all studies show the highly significant positive associations between grade and c-*erb*B-2 oncogene expression that were reported by Garcia *et al.* (1989), Paik *et al.* (1990) and Berger *et al.* (1988). However, where examined, an association that just achieves statistical significance is often found (Wright *et al.*, 1989*a*; Barnes *et al.*, 1988; Parkes *et al.*, 1990; Walker *et al.*, 1989) or alternatively there may be a non significant trend in the same direct (van de Vivjer *et al.*, 1988; Ro *et al.*, 1989). Three of the four papers published in this edition of the Journal have considered this association and the results fit the described pattern: Lovekin *et al.* describe a highly significant association ($P < 0.001$) whilst Gullick *et al.* describe the same association but with a *P*-value of only 0.04; O'Reilly on the other hand describes an association which fails to achieve statistical significance ($P = 0.12$). The story is very similar for hormone receptor status although this has been less widely evaluated. Papers describing a strong negative association between c-*erb*B-2 expression and ER or progesterone receptor status include those by Borg *et al.* (1990), Adnane *et al.* (1989), Guerin *et al.* (1989), Tandon *et al.* (1989); whilst Zeillinger *et al.* (1989), Berger *et al.* (1988), Slamon *et al.* (1987), Garcia *et al.* (1989) and May *et al.* (1990) all describe a weaker negative association. Of the papers considered in this editorial only Lovekin *et al.* describe a strong negative association between c-*erb*B-2 expression and ER status ($P < 0.003$) and this is confined to the subset of 180 patients, with stage III or IV breast cancer, no significant relationship having been seen in the group of 497 patients with operable disease.

There has been relatively little investigation of the relationship between c-*erb*B-2 expression and other markers of differentiation. Ploidy has previously been investigated by two groups neither of whom found any significant association (Tavassoli *et al.*, 1989; Ro *et al.*, 1989) although in the paper by Ro *et al.* (1989) there was a non significant trend towards an increased incidence of c-*erb*B-2 expression in aneuploid tumours (27% *vs* 14% – $P = 0.19$). O'Reilly *et al.* (1991) describe a similar association with 20% of aneuploid tumours expressing c-*erb*B-2 compared to only 9% diploid tumours ($P = 0.1$). The same authors have also extended their investigation to include not only ploidy but also S-phase fraction, and have found, in a chi-squared analysis, that c-*erb*B-2 expression was significantly higher in those tumours with an S-phase fraction above the median (25% *vs* 6% – $P = 0.003$). The correlation coefficient for the above association was, however, only 0.18 indicating that this association was weak with, less than 5% of the variability in S-phase fraction being due to it's association with c-*erb*B-2. Bacus *et al.* (1990) have performed a more detailed analysis of this association using the Feulgen DNA staining method to evaluate the DNA content of cells which were simultaneously stained for c-*erb*B-2 using a polyclonal antiserum. Forty-five cases were examined; interestingly the level of c-*erb*B-2 positivity was rather higher at 49% which might be partly explained by the inclusion of five cases of ductal carcinoma *in situ* and of other tumours with an extensive intraduct component. All 22 tumours positive for c-*erb*B-2 were found to have a near tetraploid DNA content with a mean DNA index of $1.9 \pm 0.19$ whilst the DNA content of tumours negative for c-*erb*B-2 varied from diploid to three times the normal DNA content with a mean DNA index of 1.4 ($\pm 0.58$).

The all-important relationship between c-*erb*B-2 expression and prognosis has been extremely widely investigated with differing results. One problem with the interpretation of such studies is that the characteristics of the patients included varies considerably. Some studies include only patients with operable early stage breast cancer, whilst others also include patients with stage III and IV disease. Some studies restrict their analyses to node positive and others to node negative patients. Most studies present survival data, but not all of these also present recurrence data.

Much of the initial interest in c-*erb*B-2 as a prognostic marker was generated by work of Slamon *et al.* (1987) which showed that in 86 node positive patients survival was significantly worse for those patients whose tumours were c-*erb*B-2 positive; similar results were also seen with respect to recurrence. Multivariate analysis of these data showed the independent prognosticators for survival to be the number of positive nodes, c-*erb*B-2 status, and ER status. These data were subsequently confirmed in a larger study from the same group (Slamon *et al.*, 1989) which included 526 patients, of whom 345 were node positive. No data are given for the association between c-*erb*B-2 expression and outcome for the group as a whole but in the node positive subset there was an association between c-*erb*B-2 expression and poor prognosis in terms of recurrence ($P = 0.01$) and survival ($P = 0.041$). In multivariate analysis c-*erb*B-2 expression retained its prognostic significance being second only to the number of axillary nodes in both the disease free and overall survival analysis. In the node negative subset however there was no association between c-*erb*B-2 expression and either recurrence or survival.

At least 20 other groups have now published data concerning the prognostic significance of c-*erb*B-2, of these only four groups have failed to find a prognostic effect of c-*erb*B-2 in at least one sub group (Zhou *et al.*, 1989; Ali *et al.*, 1988; Gusterson *et al.*, 1988; Barnes *et al.*, 1988). Van de Vivjer *et al.* (1988) found an effect of borderline statistical significance on a survival but not on a recurrence free survival analysis, this effect disappeared on multivariate analysis and it was concluded that c-*erb*B-2 status was of limited prognostic value. On the other hand four groups have been able to show an overall prognostic effect of c-*erb*B-2 in both recurrence free and survival analyses (Wright *et al.*, 1989*a*; Paik *et al.*,

1990; Walker *et al*., 1989; Tsuda *et al*., 1989). All of these papers show that the prognostic effect of c-*erb*B-2 is maintained in multivariate analysis. If the details of those studies which show no, or only a limited, prognostic effect of c-*erb*B-2 are compared to those where there is an overall prognostic effect it is apparent that significant prognostic effects tend to be seen in the larger studies. This is borne out by the result of the studies published in this edition of the Journal three of which showed an overall effect of c-*erb*B-2 on prognosis and all of these studies contained more than 450 patients.

Of the papers published in this edition of the Journal, only that from Gullick *et al*. shows a significant overall association between c-*erb*B-2 expression and both recurrence and survival which is confirmed in multivariate anlaysis. This paper lends further support to the hypothesis that some of the apparent differences in the prognostic significance of c-*erb*B-2 expression between groups may simply be a function of study size. In their overview they include two studies which individually showed no significant prognostic effect of c-*erb*B-2 expression, although both showed a non significant trend towards poor prognosis for patients with tumours positive for c-*erb*B-2 (Gusterson *et al*., 1988; Barnes *et al*., 1988). However, when considered together with a third study, which had individually shown an independent prognostic effect in terms of both relapse and survival (Wright *et al*., 1989*a*) the strong independent prognostic effect for the group as a whole was confirmed and shown to be equivalent in both node positive and node negative patients.

Of the other two large studies published in this edition of the *Journal*, both restrict their analyses to survival and both report a highly significant association between c-*erb*B-2 expression and poor survival. In the Lovekin paper c-*erb*B-2 is found not to be an independent prognostic indicator in a model which also considers axillary nodal stage, size of primary tumour, and histological grade. However, in this study there was a very strong association between c-*erb*B-2 status and grade and when grade was omitted from the multivariate analysis c-*erb*B-2 status became an independent prognosticator. In the Winstanley paper multivariate analysis showed c-*erb*B-2 receptor status to be an independent prognosticator alongside axillary nodal status and primary tumour size, the effect of histological grade was however not tested in this study.

Other studies have shown a significant effect of c-*erb*B-2 expression in the overall survival analysis but not in the recurrence free analysis (Paik *et al*., 1990; Parkes *et al*., 1990; Van de Vivjer *et al*., 1988), but only that by Paik shows c-*erb*B-2 expression to be independent prognostic significance on multivariate analysis.

Most groups have examined the prognostic significance of c-*erb*B-2 expression in sub-groups defined by conventional prognostic indicators. The results are again conflicting. Some papers find the prognostic effect of c-*erb*B-2 expression to be restricted to the node positive subset (Slamon *et al*., 1989; Tandon *et al*., 1989; Borg *et al*., 1990) and similar results are described by O'Reilly *et al*. who, however, show that the independent prognostic effect of c-*erb*B-2 expression was restricted to the recurrence free survival analysis. Other groups have made the potentially very important observation that there is a significant prognostic effect of c-*erb*B-2 expression in groups that would generally be considered to have a good prognosis. This includes the node negative subset where an association between c-*erb*B-2 expression and poor survival is described in papers by Wright *et al*. (1989*a*) and Ro *et al*. (1989). Paik *et al*. (1990) demonstrated that the maximum prognostic effect of c-*erb*B-2 expression, in terms of survival, was seen in those patients who had well differentiated tumours, particularly in those who were also node negative. A similar effect was noted in a study of 79 node negative patients (Richner *et al*., 1990) where although there was no overall association between c-*erb*B-2 expression and overall survival, such an association was found in the ER positive subset ($P = 0.001$). Wright *et al*. (1989*a*) also found that the prognostic effect of c-*erb*B-2 expression was stronger in ER positive than ER negative tumours and also stronger in EGFR negative tumours than EGFR positive tumours. May *et al*. (1989) have shown that it is possible to define a group of ER positive patients who have a poor prognosis. These patients, termed $ER^+$(R2), have a ratio of ER protein to, ER mRNA of greater than 1.5. These authors have recently extended this work (May *et al*., 1990) and shown that in a multivariate analysis, c-*erb*B-2 expression is independent of $ER^+$(R) status and is therefore capable of defining groups with a higher risk of relapse from within both the $ER^+$(R1) and $ER^+$(R2) groups, as well as from within the ER negative group where the effect was particularly strong.

Of the four papers published in this edition of the *Journal* Gullick *et al*. show clearly that the prognostic effect of c-*erb*B-2 receptor expression is equivalent in node positive and in node negative patients and make the extremely important point that in order to reliably demonstrate the prognostic effect of c-*erb*B-2 expression in the node negative subset large numbers of patients are required. This is because c-*erb*B-2 is expressed relatively infrequently and because node negative patients have a much better prognosis than node positive patients and therefore relapse and die less frequently. Many more node negative cases are therefore required to obtain similar statistical significance because statistical power is dependent upon the number of events in the study.

It is not clear how c-*erb*B-2 expression exerts its prognostic effect and is may be simply that tumour cells positive for c-*erb*B-2 have a growth advantage over cells negative for this oncogene. There may however also be some relationship between c-*erb*B-2 expression and responsiveness to treatment. A number of studies show that the prognostic effect of c-*erb*B-2 expression is stronger for survival than it is for recurrence (O'Reilly *et al*., 1991; Gullick *et al*., 1991; Wright *et al*., 1989*a*; Paik *et al*., 1990; Ro *et al*., 1989; Parkes *et al*., 1990; Tsuda *et al*., 1989; Van de Vivjer *et al*., 1988). This implies that part of the prognostic effect of c-*erb*B-2 expression is exerted during the period after recurrence, a period when the patients are likely to be receiving chemo- or hormonal therapy. Some preliminary results published in abstract form lend some support to this hypothesis. Fifty-nine patients with operable breast cancer who relapsed after primary surgical management alone were all treated with tamoxifen, 19 patients responded, 18 of whom (95%) had tumours that were negative for c-*erb*B-2; of the non responders, only 28 of the 40 (70%) were negative for c-*erb*B-2 ($P < 0.07$). The 13 patients who were positive for c-*erb*B-2 had a particularly poor prognosis and 12 (92%) showed evidence of disease progression within 6 months of starting tamoxifen compared to only 31 of 46 (67%) who were negative for c-*erb*B-2, post relapse survival was also significantly shorter for this group of patients ($P < 0.01$) (Wright *et al*., 1989*b*).

In summary, c-*erb*B-2 expression appears to be a useful addition to the prognostic armamentarium. There is evidence to suggest that its expression is more frequent in tumours of advanced stage and in tumours that are more poorly differentiated. These associations are however far from absolute as is shown by the marked difference in results between published papers. Because the oncogene is expressed relatively infrequently, larger studies such as those published in this edition of the *Journal* are required to clearly demonstrate its prognostic significance, and this is particularly the case when subset analyses are performed looking at those groups which would be considered to have a good prognosis by conventional prognostic indicators.

One major limitation of the studies published to date is that they are all retrospective and the patients included are therefore limited by the availability of material and the analyses limited by the availability of information regarding other prognosticators. Large prospective studies are now required to fully assess the prognostic significance of this interesting new biological variable. Such studies should also be able to provide information as the whether c-*erb*B-2 expression can predict responsiveness to chemo- or hormonal-therapy. Further work is also required to assess the relative strength of c-*erb*B-2 as a prognostic marker relative to other promising new markers such as the proteases Cathepsin D (Spyratos *et al.*, 1989; Thorpe *et al.*, 1989; Tandon *et al.*, 1990) and urokinase type plasminogen activator antigen (Janicke *et al.*, 1989) as well as other markers such as the NM23 gene (Bevilacqua *et al.*, 1989) and mutant p53 (Harris *et al.*, 1990).

### References

ADNANE, J., GAUDRAY, P., SIMON, M.P., SIMONY-LAFONTAINE, J., JEANTEUR, P. & THEILLET, C. (1989). Proto-oncogene amplification and human breast tumour phenotype. *Oncogene*, **4**, 1389.

ALI, I.U., CAMPBELL, G., LIDEREAU, R. & CALLAHAN, R. (1988) Lack of evidence for the prognostic significance of c-*erb*B-2 amplification in human breast carcinoma. *Oncogene Res.*, **3**, 139.

BACUS, S.S., BACUS, J.W., SLAMON, D.J. & PRESS, M.F. (1990). HER-2/neu oncogene expression and DNA. Ploidy analysis in breast cancer. *Arch. Pathol. Lab. Med.*, **114**, 164.

BARNES, D.M., LAMMIE, G.A., MILLIS, R.R., GULLICK, W.L., ALLEN, D.S. & ALTMAN, D.G. (1988). An immunohistochemical evaluation of c-*erb*B-2 expression in human breast carcinoma. *Br. J. Cancer*, **58**, 448.

BERGER, M.S., LOCHER, G.W., SAURER, S. & 4 others (1988). Correlation of the c-*erb*B-2 gene amplifications and protein expression in human breast carcinoma with nodal status and nuclear grading. *Cancer Res.*, **48**, 1238.

BEVILACQUA, G., SOBEL, M.E., LIOTTA, L.A. & STEEG, P.S. (1989). Association of Nm23 RNA levels in human primary infiltrating ductal breast carcinomas with lymphnode involvement and other histopathological indicators of high metastatic potential. *Cancer Res.*, **49**, 5185.

BORG, A., TANDON, A.K., SIGURDSSON, H. & 5 others (1990). HER-2/neu amplification predicts poor survival in node-positive breast cancer. *Cancer Res.*, **50**, 4332.

CLARK, G.M., DRESSLER, L.G., OWENS, M.A., POUNDS, G., OLDAKER, T. & MCGUIRE, W.L. (1989). Prediction of relapse or survival in patients with node-negative breast cancer by DNA flow cytometry. *N. Engl. J. Med.*, **320**, 627.

COUSSENS, L., YANG-FENG, T.L., LIAO, Y.C. & 9 others (1985). Tyrosine Kinase receptor with extensive homology to EFG receptor shares chromosomal location with *neu* oncogene. *Science*, **230**, 1132.

EARLY BREAST CANCER TRIALISTS COLLABORATIVE GROUP (1988). Effects of adjuvant tamoxifen and of cytotoxic therapy on mortality in early breast cancer. An overview of 61 randomized trials among 28,896 women. *N. Engl. Med.*, **319**, 1681.

FALLENIUS, A.G., FRANZEN, S.A. & AUER, G.U. (1988). Predictive value of nuclear DNA content in breast cancer in relation to clinical and morphologic factors. A retrospective study of 227 consecutive cases. *Cancer*, **62**, 521.

FISHER, B., BOWER, M., MARGOLESE, R. & 16 others (1985). Five-year results of a randomized clinical trial comparing total mastectomy and segmental mastectomy with or without radiation in the treatment of breast cancer. *N. Engl. J. Med.*, **312**, 665.

FISHER, B., REDMOND, C., FISHER, E.R., CAPLAN, R. & OTHER CONTRIBUTING NATIONAL SURGICAL ADJUVANT BREAST AND BOWEL PROJECT INVESTIGATORS (1988). Relative worth of estrogen or progesterone receptor and pathologic characteristics of differentiation as indicators of prognosis in node negative breast cancer patients; findings from National Surgical Adjuvant Breast and Bowel project protocol B-06. *J. Clin. Oncol.*, **6**, 1076.

GARCIA, I., DIETRICH, P., AAPRO, M., VAUTHIER, G., VADAS, L. & ENGEL, E. (1989). Genetic alterations of c-*myc*, c-*erb*B-2, and c-Ha-*ras* proto-oncogenes and clinical associations in human breast carcinomas. *Cancer Res.*, **49**, 6675.

GUERIN, M., GABILLOT, M., MATHIEU, M.C. & 4 others (1989). Structure and expression of c-*erb*B-2 and EGF receptor genes in inflammatory and non-inflammatory breast cancer: prognostic significance. *Int. J. Cancer*, **43**, 201.

GULLICK, W.J., BERGER, M.S., BENNETT, P.L.P., ROTHBARD, J.B. & WATERFIELD, M.D. (1987). Expression of the c-erbB-2 protein in normal and transformed cells. *Int. J. Cancer*, **40**, 246.

GULLICK, W.J., LOVE, S.B., WRIGHT, C. & 4 others (1991). c-*erb*B-2 protein overexpression in breast cancer is a risk factor in patients with involved and uninvolved lymph nodes. *Br. J. Cancer*, **63**, 434.

GUSTERSON, B.A., MACHIN, L.G., GULLICK, W.J. & 6 others (1988). c-*erb*B-2 expression in benign and malignant breast disease. *Br. J. Cancer*, **58**, 453.

HAYBITTLE, J.L., BLAMEY, R.W., ELSTON, C.W. & 5 others (1982). A prognostic index in primary breast cancer. *Br. J. Cancer*, **45**, 361.

HARRIS, A.L., HORAK, E., SMITH, K. & 4 others (1990). Mutant p53 a common genetic abnormality in human breast cancer and associated with EGF receptor and neu expression. *Br. J. Cancer*, **62**, 503.

HENDERSON, I.C., HARRIS, J.R., KINNE, D.W. & HELLMAN, S. (1989). Cancer of the breast. In *Cancer Principals & Practice of Oncology*, deVita, B.T., Hellman, S. & Rosenberg, S.A. (eds) p. 1208. J.B. Lippincott: Philadephia.

JANICKE, F., SCHMITT, M., ULM, K., GOSSNER, W. & GRAEFF, H. (1989). Urokinase-type plasminogen activator antigen and early relapse in breast cancer. *Lancet*, **ii**, 1049.

LOVEKIN, C., ELLIS, I.O., LOCKER, A. & 6 others (1991). c-*erb*B-2 oncoprotein expression in primary and advanced breast cancer. *Br. J. Cancer*, **63**, 439.

O'REILLY, S.M., BARNES, D.M., CAMPLEJOHN, R.S., BARTKOUA, J., GREGORY, W.M. & RICHARDS, M.A. (1991). The relationship between c-*erb*B-2 expression, S-phase fraction and prognosis in breast cancer. *Br. J. Cancer*, **63**, 444.

MAY, E., MOURIESSE, M., MAY-LEVIN, F., CONTESSO, G. & DELARUE, J.C. (1989). A new approach allowing an early prognosis in breast cancer; a ratio of oestrogen receptor (ER) ligand binding activity to the ER-specific mRNA level. *Oncogene*, **4**, 1037.

MAY, E., MOURIESSE, H., MAY-LEVIN, F., QIAN, J.F., MAY, P. & DELARUE, J.C. (1990). Human breast cancer; identification of populations with a high risk of early relapse in relation to both oestrogen receptor status and c-*erb*B-2 over-expression. *Br. J. Cancer*, **62**, 430.

NOLVADEX ADJUVANT TRIAL ORGANISATION (1988). Controlled trial of tamoxifen as a single adjuvant agent in the management of early breast cancer. *Br. J. Cancer*, **57**, 608.

PAIK, S., HAZAN, R., FISHER, E.R. & 6 others (1990). Pathological findings from the National Surgical Adjuvant Breast and Bowel Project: prognostic significance of erbB-2 protein overexpression in primary breast cancer. *J. Clin. Oncol.*, **8**, 103.

PARKES, H.C., LILLYCROP, K., HOWELL, A. & CRAIG, R.K. (1990). c-*erb*B-2 mRNA expression in human breast tumours: comparison with c-*erb*B-2 DNA amplification and correlation with prognosis. *Br. J. Cancer*, **61**, 39.

RICHNER, J., GERBER, H.A., LOCHER, G.W. & 6 others (1990). c-*erb*B-2 protein expession in node negative breast cancer. *Ann. Oncol.*, **1**, 263.

RO, J., EL-NAGGAR, A., RO, J.Y. & 5 others (1989). c-*erb*B-2 amplification in node negative human breast cancer. *Cancer Res.*, **49**, 6941.

SAINSBURY, J.R.C., FARNDON, J.R., NEEDHAM, G.K., MALCOLM, A.J. & HARRIS, A.L. (1987). Epidermal-growth-factor receptor status as predictor of early recurrence of and death from breast cancer. *Lancet*, **i**, 1398.

SLAMON, D.J., CLARK, G.M., WONG, S.G., LEVIN, W.J., ULLRICH, A. & MCGUIRE, W.L. (1987). Human breast cancer: correlation of relapse and survival with amplification of the HER-2/neu oncogene. *Science*, **235**, 177.

SLAMON, D.J., GODOLPHIN, W., JONES, L.A. & 8 others (1989). Studies of the HER-2/neu proto-oncogene in human breast and ovarian cancer. *Science*, **244**, 707.

SPYRATOS, F., MAUDELONDE, T., BROUILLET, J. & 7 others (1989). Cathepsin D; an independent prognostic factor for metastasis of breast cancer. *Lancet*, **ii**, 1115.

TANDON, A.K., CLARK, G.M., CHAMNESS, G.C., ULLRICH, A. & MCGUIRE, W.L. (1989). HER-2/neu oncogene protein and prognosis in breast cancer. *J. Clin. Oncol.*, **7**, 1120.

TANDON, A.K., CLARK, G.M., CHAMNESS, G.C., CHIRGWIN, J.M. & MCGUIRE, W.L. (1990). Cathepsin D and prognosis in breast cancer. *N. Engl. J. Med.*, **322**, 297.

TAVASSOLI, M., QUIRKE, P., FARZANEH, F., LOCK, N.J., MAYNE, L.V. & KIRKHAM, N. (1989). c-*erb*B-2/c-*erb*A co-amplification indicative of lymph node metastasis, and c-myc amplification of high tumour grade, in human breast carcinoma. *Br. J. Cancer*, **60**, 505.

THOR, A.D., SCHWARTZ, L.H., KOERNER, F.C. & 8 others (1989). Analysis of c-erbB-2 expression in breast carcinomas with clinical follow-up. *Cancer Res.*, **49**, 7147.

THORPE, S.M., ROCHEFORT, H., GARCIA, M. & 7 others (1989). Association between high concentrations of $M_r$ 52,000 Cathepsin D and poor prognosis in primary human breast cancer. *Cancer. Res.*, **49**, 6008.

TODD, J.H., DOWLE, C., WILLIAMS, M.R. & 5 others (1987). Confirmation of a prognostic index in primary breast cancer. *Br. J. Cancer*, **56**, 489.

TSUDA, H., HIROHASHI, S., SHIMOSATO, Y. & 11 others (1989). Correlation between long-term survival in breast cancer patients and amplification of 2 putative oncogene-coamplification units; hst-1/int-2 and c-erbB-2/ear-1. *Cancer Res.*, **49**, 3104.

VAN DER SCHUEREN. E. & VAN DONGEN, J.A. (1988). Management of early breast cancer – current status of treatment; workshop report. *Eur. J. Cancer Clin. Oncol.*, **24**, 89.

VAN DE VIVJER, M.J., PETERSE, J.L., MOOI, W.J. & 4 others (1988). Neu-protein overexpression in breast cancer. Association with comedo-type ductal carcinoma in situ and limited prognostic value in stage II breast cancer. *N. Engl. J. Med.*, **319**, 1239.

VERONESI, U., BANFI, A., SALVADORI, B. & 11 others (1990). Breast conservation is the treatment of choice in small breast cancer: long term results of a randomized trial. *Eur. J. Cancer*, **26**, 668.

WALKER, R.A., GULLICK, W.J. & VARLEY, J.M. (1989). An evaluation of immunoreactivity for c-erbB-2 protein as a marker of poor short-term prognosis in breast cancer. *Br. J. Cancer*, **60**, 426.

WINSTANLEY, J., COOKE, T., MURRAY, G.D. & 7 others (1991). The long term prognostic significance of c-*erb*B-2 in primary breast cancer. *Br. J. Cancer*, **63**, 447.

WRIGHT, C., ANGUS, B., NICHOLSON, S. & 6 others (1989*a*). Expression of c-*erb*B-2 oncoprotein: a prognostic indicator in human breast cancer. *Cancer Res.*, **49**, 2087.

WRIGHT, C., NICHOLSON, S., ANGUS, B. & 5 others (1989*b*). Association of c-*erb*B-2 oncoprotein expression with lack of response to endocrine therapy in recurrent breast cancer. *J. Pathol.*, **158**, 350A.

ZEILLINGER, R., KURY, F., CZERWENKA, K. & 11 others (1989). HER-2 amplification, steroid receptors and epidermal growth factor receptor in primary breast cancer. *Oncogene*, **4**, 109.

ZHOU, D.J., AHUJA, H. & CLINE, M.J. (1989). Proto-oncogene abnormalities in human breast cancer: c-*erb*B-2 amplification does not correlate with recurrence of disease. *Oncogene*, **4**, 105.